# “It’s Totally Okay to Be Sad, but Never Lose Hope”: Content Analysis of Infertility-Related Videos on YouTube in Relation to Viewer Preferences

**DOI:** 10.2196/10199

**Published:** 2018-05-23

**Authors:** Margot Kelly-Hedrick, Paul H Grunberg, Felicia Brochu, Phyllis Zelkowitz

**Affiliations:** ^1^ Department of Psychiatry Jewish General Hospital Montreal, QC Canada; ^2^ Department of Psychology McGill University Montreal, QC Canada; ^3^ Department of Psychiatry McGill University Montreal, QC Canada; ^4^ Lady Davis Institute for Medical Research Montreal, QC Canada

**Keywords:** infertility, internet, YouTube, social media, online health information

## Abstract

**Background:**

Infertility patients frequently use the internet to find fertility-related information and support from people in similar circumstances. YouTube is increasingly used as a source of health-related information and may influence health decision making. There have been no studies examining the content of infertility-related videos on YouTube.

**Objective:**

The purpose of this study was to (1) describe the content of highly viewed videos on YouTube related to infertility and (2) identify video characteristics that relate to viewer preference.

**Methods:**

Using the search term “infertility,” the 80 top-viewed YouTube videos and their viewing statistics (eg, views, likes, and comments) were collected. Videos that were non-English, unrelated to infertility, or had age restrictions were excluded. Content analysis was used to examine videos, employing a coding rubric that measured the presence or absence of video codes related to purpose, tone, and demographic and fertility characteristics (eg, sex, parity, stage of fertility treatment).

**Results:**

A total of 59 videos, with a median of 156,103 views, met the inclusion criteria and were categorized into 35 personal videos (35/59, 59%) and 24 informational-educational videos (24/59, 41%). Personal videos did not differ significantly from informational-educational videos on number of views, dislikes, subscriptions driven, or shares. However, personal videos had significantly more likes *(P*<.001) and comments *(P<*.001) than informational-educational videos. The purposes of the videos were treatment outcomes (33/59, 56%), sharing information (30/59, 51%), emotional aspects of infertility (20/59, 34%), and advice to others (6/59, 10%). The tones of the videos were positive (26/59, 44%), neutral (25/59, 42%), and mixed (8/59, 14%); there were no videos with negative tone. No videos contained only male posters. Videos with a positive tone did not differ from neutral videos in number of views, dislikes, subscriptions driven, or shares; however, positive videos had significantly more likes (*P<*.001) and comments (*P<*.001) than neutral videos. A majority (21/35, 60%) of posters of personal videos shared a pregnancy announcement.

**Conclusions:**

YouTube is a source of both technical and personal experience-based information about infertility. However, videos that include personal experiences may elicit greater viewer engagement. Positive videos and stories of treatment success may provide hope to viewers but could also create and perpetuate unrealistic expectations about the success rates of fertility treatment.

## Introduction

Up to 1 in 6 Canadian couples may experience infertility [1], defined as the failure to achieve conception following at least 12 months of unprotected intercourse [2]. Prevalence rates are similar or higher in the United States and European countries, with estimates ranging from 10% to 28% [3-7]. The diagnosis and treatment of infertility may have adverse effects on psychological well-being and quality of life [8-10].

More than 85% of infertility patients search for Web-based infertility-related content [11-13]. They report using the Web to find experience-based information; to learn about others going through similar treatment [14]; and to gain emotional support, reduce isolation, and seek normalcy [15]. The internet is an increasingly common source of health-related information, with 72% of internet users using the Web for health information [16].

Social media allows internet users to connect with others online, facilitating communication and social support regarding health conditions [17]. YouTube is one such social media website that allows users to share video content and engage in discussion. YouTube is the most popular website for video sharing and the second most accessed website with over 1 billion users, reaching a global and diverse audience [18,19]. YouTube is a source of experience-based information on health topics [20,21] and the use of YouTube for social support relating to health topics has been found to relate to higher levels of health care–related empowerment and information engagement [22]. Therefore, examining the content of popular health-related videos on YouTube is of interest. Several studies have examined how YouTube videos characterize chronic health conditions, such as epilepsy [23], Alzheimer's disease [24], and chronic obstructive pulmonary disease [25]. These studies have evaluated level of viewership, discussion, and reliability of information presented in videos on YouTube [23,25,26]. There has not yet been an examination of infertility content on YouTube. As such, this is the first study to assess highly viewed infertility videos on YouTube to document their tone, purpose, and characteristics of posters and fertility-related variables, using content analysis. The coding scheme was based on other studies examining health-related content on YouTube [23,25-28]. The study aimed to (1) describe the video content of the most highly viewed videos and (2) identify video characteristics that relate to viewer favorability (eg, video likes, shares). Doing so may provide a richer understanding of people’s infertility experiences and purpose for posting infertility information on the internet. Indications of viewers’ responses to videos, such as likes and dislikes, may give an indication of viewer preference.

## Methods

### Ethical Considerations

YouTube meets the criteria for a public online database as it is free, publicly accessible without requiring registration, and has a large membership size [18,29]. Consistent with past research [29,30], our institutional ethics board deemed ethics approval unnecessary as content relevant to this study was in the public domain. YouTube was accessed without a registered account to ensure accessed videos were publicly available and had no age restrictions.

### Coding Rubric Development

The coding rubric for the video analysis was initially developed through a deductive approach. Specifically, we reviewed studies that used content analysis to examine health-related YouTube topics [23,25-28]. Videos were coded as either personal, where the primary focus is on sharing experiences, or informational-educational, where the focus is to provide information. Other variables included (1) tone (positive, negative, neutral, or mixed), (2) purpose(s) of video (sharing information, emotional aspects of infertility, treatment outcomes, and advice to others), (3) whether the video has live people present (character video) or absent (noncharacter video), and (4) source of video, which indicates the type of YouTube channel that posted the video (personal account, organization’s channel, news channel, or other type of channel). Before viewing videos, the coding team generated categories hypothesized to be relevant to infertility-related videos, such as the stage of fertility treatment of the poster(s) and parity of the poster(s). Two coders tested the rubric using 7 sets of 3 to 5 videos with low view counts. Over the course of this testing period, the codebook was modified until variables were refined and explicitly defined. Through an inductive process, new variables such as the code “pregnancy announcement” were added if agreed upon by both coders during the training process. Training videos were not included in the final dataset. The training period ended when the 2 coders reached agreement for all codes and code definitions (see Table 1 for codebook). Certain variables (purpose of video, type of informational video, and subtones in videos) were not mutually exclusive, and therefore codes could sum to more than 100%. Video statistics were collected for all videos in the sample. These included number of views, likes, dislikes, comments, date video was posted, transcript (generated by YouTube), subscriptions driven (number of people who subscribed to the channel following viewing of video), number of shares, length of video, number of subscribers to channel, account creation date, and total channel views (sum of views for all videos posted by user). YouTube channels are profiles for users to post their videos and share information about themselves and their videos.

**Table 1:** Codebook variable definitions and inter-rater reliability for 2 coders assessed by Cohen kappa.

Variable definitions			Kappa^a^
**Variables coded for all video types**			
	**Type of video**		1.00
		Personal**:** Primary focus on sharing experiences, stories, or emotions	
		Informational-educational: Primary focus is to provide information	
	**Character and noncharacter videos**		1.00
		Character: Contains 1 or more live people	
		Noncharacter: Contains no live people in video (eg, animation or slideshow of photos)	
	**Purpose for posting**		
		Sharing information: Information, facts, details and descriptions of infertility conditions, causes, and treatments. May include information that poster finds online, in books, or by reading scientific papers	1.00
		Emotional aspects of infertility: Discussion of the emotional aspects of infertility process, diagnosis and/or treatment (ie, how individual or couple feels, processing of emotions)	.71
		Treatment outcomes: Discussion of treatment outcome(s). May include infertility treatment experiences (ie, reporting positive or negative treatment results)	1.00
		Advice to others: Individual or couple in video is giving advice to others; advocating for a treatment, decision, or action	.75
	**Source of video**		.63
		Personal account: An individual’s (or couple’s) channel	
		Organization’s channel: An institute, organized group, or company’s channel	
		News channel: A news source’s channel	
		Other: Type of channel is unclear or does not fit other categories	
	**Source: Type of organization**		.77
		Drug company: Channel belongs to a drug or pharmaceutical company	
		Doctor or clinic: Channel belongs to a medical clinic or a doctor	
		Academic institution: Channel belongs to a university, medical school, or other education-promoting channel	
		Other: Channel belongs to organization, but does not fit into another category	
	**Tone of video**		1.00
		Positive: More than 50% of video tone is positive, indicated by the dominance of 1 or more of the positive subtones (see below)	
		Negative: More than 50% of the video tone is negative, indicated by the dominance of 1 or more of the negative subtones (see below)	
		Neutral: Absence of overarching positive or negative tones	
		Mixed: The video tone is approximately 50% positive and 50% negative	
	**Positive subtones in video**		
		Hope: Overall sentiment that their future holds improvements or success, even in the face of current adversities	.69
		Encouraging: Reassurance and hope toward viewers/others; offers statements of inspiration, comfort, and/or hope	.85
		Humorous: Humor or jokes when talking about their treatment or throughout the video	.83
		Joyful: Laughing, excited comments, and joyous exclamations	.83
		Grateful: Thankfulness, gratitude, or feeling blessed for life/situation	.84
	**Negative subtones in video**		
		Anger or frustration: Anger or frustration toward self, others, or situation	—^b^
		Sadness: Sorrow evidenced by verbal statements and/or frowning, crying, downcast expression	—^b^
		Hopelessness: An overall sentiment that things will not get better	—^b^
	**Thanking viewers**		
		Thanking viewers for providing support or participation on YouTube (ie, subscriptions, comments)	1.00
**Variables coded for informational-educational videos only**			
	**Type of informational-educational video**		
		Infertility causes or detection: Causes, signs, or detection of infertility	.88
		Medical procedures: How a medical procedure works (eg, in vitro fertilization) or how a lab technique works (eg, measuring sperm motility)	.89
		Lifestyle: Lifestyle choices (ie, diet, exercise, sleep)	1.00
		Psychological: Mental health factors relating to infertility	.63
**Variables coded for personal videos only**			
	**Relationship of individual(s) in video**		.87
		Romantic or unspecified: Explicitly states being in a relationship, but marital status not specified	
		Engaged: States being engaged	
		Married: States being married	
		Single: States being single	
		Missing: Does not explicitly state relationship or unclear	
	**Relationship type**		.86
		Male and female, female and female, male and male, male only (ie, single male), female only (ie, single female), or unclear from video if individuals in video are partnered	
	**Sex of people in video**		1.00
		Going through treatment together and in video: Male and female, female and female, male and male, male only (ie, single), female only (ie, single), or unclear from video	
	**Parity**		.88
		Number of children explicated stated	
	**Stage of fertility treatment**		1.00
		Pretreatment: Have not started any fertility drugs or treatment	
		Undergoing treatment: Currently undergoing treatment, procedures, or taking medications	
		Pregnancy: Currently pregnant	
		Pregnancy failure: Failed round of treatment and no pregnancy	
		Postchildbirth: Child has been born	
		Posttreatment: Treatment completed and waiting on results	
	**Round of treatment**		1.00
		First round of treatment: Started or undergone first round	
		Multiple rounds of treatment: Started or undergone more than 1 round	
	**Live pregnancy results**		1.00
		Pregnancy results disclosed to spouse, friends, or family in video	

^a^2 coders, n=14 training videos.

^b^Codes for at least 1 coder were a constant; therefore, we were unable to calculate the kappa statistic.

### Sample

In January 2017, the most-viewed infertility YouTube videos were identified using the search term “infertility” on YouTube on Google Chrome browser with the search history and cookies cleared [31]. We chose the term “infertility” to capture the most common, basic query a patient would be likely to search, as people generally use keywords and condition names when searching for health information online [32]. This search produced approximately 314,000 results. Videos were sorted by view count and the 80 most-viewed videos were selected for analysis. Previous research suggests over 90% of users click on results in the first 3 pages of a search [33]; therefore, we elected to include the first 4 pages of results to ensure selection of popular infertility videos most likely to be encountered (20 results per page). Data for each video were collected and stored in Microsoft Excel. When variable information was not provided for a video (eg, number of shares), it was coded as missing. The URL and title of video were saved as a reference during data analysis.

**Figure 1:**
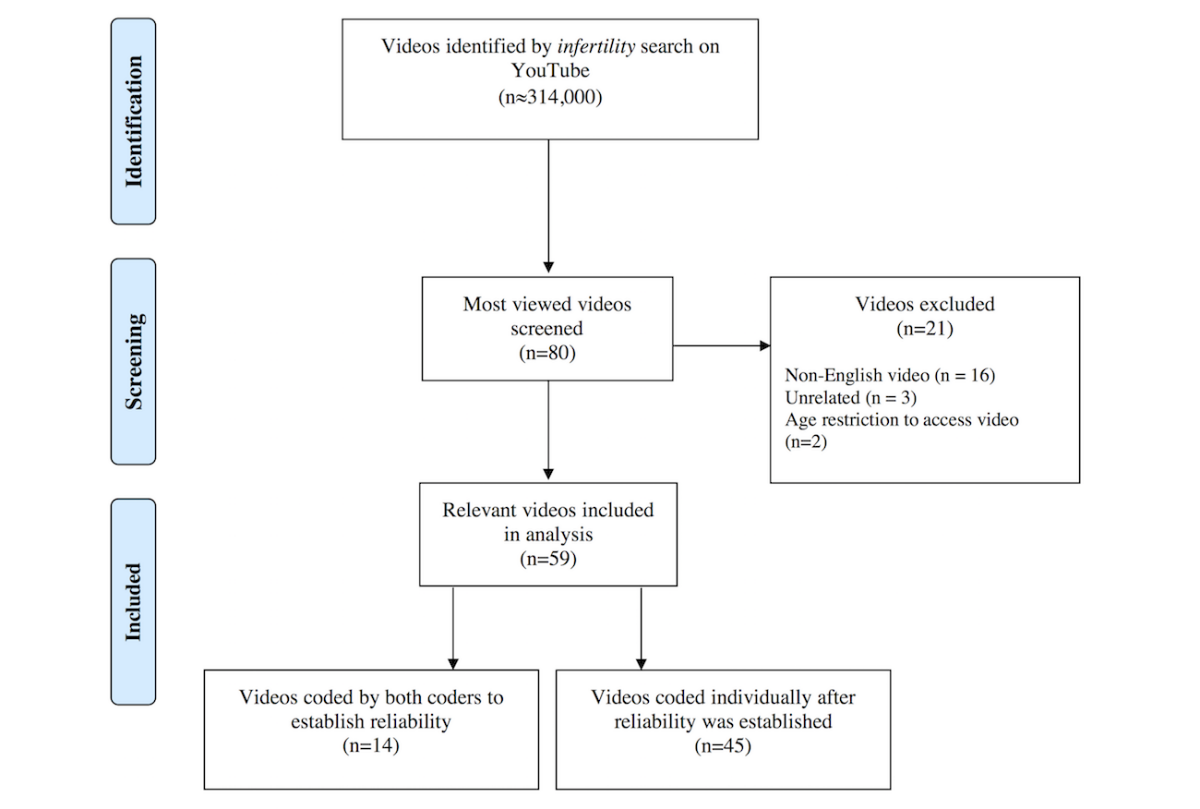
Diagram of selection process of YouTube videos for inclusion in sample.

Videos were excluded from the sample if they were non-English (n*=* 16), unrelated to infertility (n*=* 3), or had age-restricted access (n=2). This produced a final dataset of 59 videos. Refer to Figure 1 for an inclusion flowchart.

Approximately 20% of the final dataset (14/59, 24%) was independently coded by 2 coders (MKH and FB) to examine inter-rater reliability. Cohen kappa was used to evaluate inter-rater reliability (see Table 1 for kappa coefficients). Kappa coefficients ranged from .638 (substantial agreement) to 1.00 (near perfect agreement), reflecting satisfactory or excellent inter-rater reliability for health-related research [34]. All analysis was performed using SPSS Statistics 23 software (IMB Analytics). Median value and inter-quartile range were used for most video statistics to provide a measure of central tendency, which tends to be less influenced by skewed data. A similar approach has been used in previous research of health-related topics on YouTube [35-37].

## Results

### Classification of Videos

The total sample of 59 videos ranged from 50 seconds to 40 min in length (mean 6.6 min, SD 7.10 min). Videos were collectively viewed almost 23 million times (median=156,103 views). Videos received more likes (median=525 likes) than dislikes (median=25.5 dislikes), with a median of 55 comments per video. Only 35 videos indicated number of shares, with a median of 105 shares per video (see Table 2 for video popularity statistics).

Videos were classified into 4 channel categories—personal accounts (23/59, 39%), organizations’ channels (20/59, 34%), news channels (2/59, 3%), and accounts that could not be classified (eg, due to lack of information on channel; 14/59, 24%). The channels had a median of almost 6000 subscribers.

**Table 2:** Median and interquartile range of video popularity measures in total video sample (n=59).

Video Statistic	n^a^	Median	Interquartile range
Views	59	156103.0	230000.0
Likes	58	525.0	1892.2
Dislikes	58	25.5	47.5
Comments	57	55.0	354.5
Shares	35	105.0	201.0
Subscribers	59	5992.0	189273.0

^a^n varies as video statistics were not available for all videos.

The majority of videos had people in them (character videos, 48/59, 81%), whereas 19% had animation or photos (noncharacter videos, 11/59). More than half of the videos discussed treatment outcomes (33/59, 56%) and half shared information (30/59, 51%). About one-third of videos discussed emotional aspects of infertility (20/59, 34%) and 10% offered advice to others (6/59). The sample contained more personal (35/59, 59%) than informational-educational (24/59, 41%) videos. The characteristics of these 2 categories of videos are discussed below and are summarized in Table 3 (personal videos) and Table 4 (informational-educational videos).

**Table 3:** Frequency and proportion of codes for personal videos (n=35).

Variable		n (%)
**Purpose for posting^a^**		
	Treatment outcomes	33 (94)
	Emotional aspects	18 (51)
	Sharing information	6 (17)
	Advice to others	4 (11)
**Overall tone**		
	Positive	26 (74)
	Mixed	8 (23)
	Neutral	1 (3)
**Source of video**		
	Personal account	23 (66)
	Organization	1 (3)
	Other	9 (26)
	News source	2 (6)
**Sex of Poster(s)**		
	Female	12 (34)
	Male-female couple	12 (34)
	More than 2 individuals in video	10 (29)
	Relationship unclear	1 (3)
**Relationship type**		
	Heterosexual	34 (97)
	Unspecified	1 (3)
**Relationship status**		
	Married	18 (51)
	Unspecified	16 (46)
	Unclear	1 (3)
**Parity**		
	No children	9 (26)
	One child	6 (17)
	More than 1 child	3 (9)
	Number of children not stated	17 (48)
**Round of treatment**		
	Unspecified	21 (60)
	Multiple rounds	13 (37)
	First round	1 (3)

^a^Videos could be coded for more than 1 purpose.

**Table 4:** Frequency and proportion of codes for informational-educational videos (n=24).

Variable			n (%)
**Purpose for posting^a^**			
	Sharing information		24 (100)
	Emotional aspects		2 (8)
	Advice to others		2 (8)
**Type of informational video^a^**			
	Infertility causes or detection		16 (67)
	Medical procedures		14 (58)
	Lifestyle		7 (29)
	Psychological		6 (25)
**Overall tone**			
	Neutral		24 (100)
**Source of video**			
	**Organization**		19 (79)
		Doctor/clinic	9 (38)
		Other organization	9 (38)
		Academic institute	1 (4)
	Other		9 (47)

^a^Videos could be coded for more than 1 purpose for posting and more than 1 type of informational video.

### Personal Videos

#### Characteristics of Posters

More than two-thirds of personal videos contained a female (12/35, 34%) or male-female couple (12/35, 34%); none of the videos contained only a male. In all but 1 video, the individual or couple was coded as being in a heterosexual relationship (34/35, 97%). Approximately half of all personal videos included couples who were married (18/35, 51%) and nearly half did not specify their relationship status (16/35, 46%). Similarly, parity was stated in about half of the videos—9 videos (9/35, 26%) reported no children, 6 videos (6/35, 17%) reported 1 child, and 3 videos (3/35, 9%) reported more than 1 child.

Only one-fifth of personal videos mentioned a specific infertility diagnosis, which included polycystic ovary syndrome and low sperm motility (7/35, 20%). Type of infertility treatment was mentioned in about half of personal videos (18/35, 51%), and included both medical (eg, ovulation medication, intrauterine insemination, in vitro fertilization, surgery) and nonmedical treatments (eg, diet, supplements, exercise, psychological treatment, alternative medicine). In almost two-thirds of personal videos (22/35, 63%), the poster was at the pregnancy stage of treatment and the remainder were in pretreatment (1/35, 3%), undergoing treatment (3/35, 9%), posttreatment (2/35, 6%), postchildbirth (6/35, 17%), or not specified (2/35, 6%). One-third of posters disclosed they had undergone multiple rounds of treatment (13/35, 37%), whereas only 1 mentioned it was their first round (1/35, 3%).

#### Purpose

A total of 33 of the personal videos (33/35, 94%) coded as “treatment outcomes” disclosed the outcome of a diagnosis, test, or treatment. For example, 1 woman filmed herself watching her pregnancy test turn positive and exclaimed:

My heart is racing, okay, let’s see. [looks at stick] Oh my god. Oh my god. It’s positive...so it’s positive.

Of these 33 videos, 21 (21/33, 64%) were pregnancy announcements that included live results of tests or sharing the results with their spouse, friends, or family.

Videos discussing how the poster felt during treatment were coded as “emotional aspects of infertility” (18/35, 51%). One woman detailed her emotional responses to the successes and failures of treatment:

I was on ovulation medication for several months and each month was an emotional rollercoaster of hoping we’d get pregnant and not being pregnant. Until finally, one month, we were pregnant...we were just so happy...unfortunately, two weeks after...I miscarried the baby. And it was such a sadness, my heart was broken. I remember crying and the pain and the fear and the emptiness and heartache.

In some cases, videos discussing emotional aspects of infertility mentioned interactions with those not struggling with infertility. One couple, reflecting on their past infertility experiences, shared:

Whatever the situation, it is okay to be sad, but it’s also important to be happy for others. And that was sometimes very...hard for me.

Similarly, some videos discussed the anxiety related to waiting for test results or preparing for treatment. One woman made a video the day she was preparing to undergo a hysterosalpingogram test and stated:

I have been having extreme bouts of anxiety and I’m...really nervous...I’m like freaking out...the anticipation kills you, you know.

Videos were coded as “sharing information” if they included information provided by doctors or from online research (6/35, 17%). One individual discussed raising awareness about infertility:

I want to be the person to tell you guys, that from a young age, we’re taught how to prevent pregnancy and how to not get pregnant for a very long time until we’re successful and have a job and have graduated college...what our parents and teachers and doctors aren’t telling us, is when you get to a certain age, it is close to impossible to have a baby without fertility treatments and IVF.

Finally, 4 personal videos (4/35, 11%) were coded as ‘advice to others,’ reflecting recommendations or advice to viewers about concrete actions or decisions they should make regarding infertility. This advice was often based on personal experience. For example, 1 woman talking about her journey to get pregnant at age 40, suggested lifestyle changes to her viewers:

I really recommend that you do the basal body temperature chart...by eating vegetables and fruits and drinking plenty of water and eliminating caffeine and alcohol, you’re putting yourself into a more pH balanced state.

#### Tone

Of the 35 personal videos, the dominant tones were: positive (26/35, 74%), mixed (8/35, 23%), and neutral (1/35, 3%). No videos were coded as having a negative tone.

Videos with a positive tone were coded for the presence of 5 positive subtones—joy (25/26, 96%), hope (5/26, 19%), encouragement (5/26, 19%), humor (5/26, 19%), and gratitude (3/26, 12%). The majority of positive videos included joy, often indicated by facial expression, joyful crying, and exclamations such as feeling “on cloud nine.” Videos with the subtone hope included statements such as:

Such build up...we just hope that we’ve got something to show for it...we hope they’re positive [the results] but it’s totally out of our control so fingers crossed for it.

Similarly, another poster discussed the decision to undergo a second round of intrauterine insemination (IUI):

I got inside my own head and I pictured everything being a success...I said, ‘no I want to do it [IUI], I feel really confident.

When those posting conveyed hope or support to the viewer, the video was coded as encouraging, exemplified by the following statement:

I think it’s totally okay to be sad, but never lose hope. Don’t ever get yourself down. I mean, we all have moments where we feel vulnerable and sad and upset and everything but let that just be a small moment of your time. Let happiness fill you.

Videos that incorporated humor included jokes and laughter. A man in 1 video bantered with a relative when disclosing a pregnancy announcement, “this time I’m not joking.” Finally, a small number of videos contained statements of gratitude. In 1 video, a couple discussing their child and their second child soon to come, stated:

We say family prayers together and we talk about what we’re grateful for...we really don’t let a day pass us that we don’t...remind ourselves...I cannot believe we have [a child]...I’m grateful to be pregnant.

A total of 11 videos (11/59, 19%) included messages thanking viewers or subscribers for watching, commenting on, liking, subscribing, or otherwise showing support to the poster.

When posters discussed both positive and negative experiences, the tone was coded as mixed. For example, 1 woman describing her infertility journey expressed frustration (ie, negative tone) with the doctors she initially encountered:

He [the doctor] was literally dismissing me because I was not old enough in his eyes to deserve treatment...that was something that was one of the hardest things that I dealt with, with my infertility. It was people not taking us seriously...Whose business was it to tell us we couldn’t [have kids yet]? And it wasn’t fair and it was frustrating and I was angry because...I felt like my right as a human being, as a woman was taken from me. It was the most frustrating, emotional, devastating thing to go through.

After switching fertility doctors and undergoing treatment, the woman was able to achieve pregnancy, making the positive statement:

I finally saw those two lines [on the pregnancy stick]...It was the greatest moment...It’s the greatest thing to achieve getting pregnant when you haven’t been able to...I will never take it for granted.

The 1 neutral video included an acupuncturist who shared her personal story on natural pregnancy, explaining the steps she took to conceive.

### Informational-Educational Videos

The purpose of all 24 informational-educational videos was coded as sharing information and each was neutral in tone. Emotional aspects of infertility treatment and advice to viewers were each present in 2 videos. Over half of the videos contained information relating to infertility causes or detection (16/24, 67%) and/or medical procedures (14/24, 58%), whereas less than one-third discussed lifestyle factors (7/24, 29%) and/or psychological factors related to infertility (6/24, 25%).

### Characteristics Relating to Video Popularity

Personal videos (mean 2507.66, SD 2663.87) had significantly more likes than informational-educational videos (mean 241.22, SD 338.38; *t*_56_=4.04; *P*<.001). Personal and informational-educational videos did not differ on number of views, dislikes, subscriptions driven, or shares. Similarly, personal videos (mean 347.32, SD 405.63) had significantly more comments than informational-educational videos (mean 33.48, SD 36.48; *t*_55_=3.69; *P<*.001).

Positive (n=26) and neutral (n=25) videos were further analyzed for features relating to video popularity. Personal videos with mixed tone (n=8) were not analyzed due to small sample size. Positive and neutral videos did not differ on number of views, dislikes, subscriptions driven, or shares. Positive videos had significantly more likes (mean 2059.56, SD 2355.67) than neutral videos (mean 254.29, SD 337.08; *t*_47_=3.79; *P*<.001). Positive videos also had significantly more comments (mean 267.32, SD 321.34) than neutral videos (mean 34.38, SD 35.95; *t*_46_=3.63; *P*<.001). As all positive videos (n*=* 26) were personal videos, it was not possible to determine whether greater engagement was due to videos being positive or personal.

## Discussion

### Principal Findings

This study was the first to examine infertility-related videos on YouTube using a content analysis method. These videos had high numbers of comments, likes, shares, and subscriptions, indicating that many individuals seek out and engage with infertility-related information on YouTube. The videos were categorized into informational-educational videos and personal videos. Personal and informational-educational videos did not differ in number of views, supporting claims that people use YouTube for both health-related information [38] and social interaction and discussion [38,39]. However, personal videos did elicit more engagement as evidenced by more likes and comments. This suggests that, compared with informational-educational videos, personal videos may resonate more strongly with viewers. One reason may be that personal videos allow people to observe others undergoing similar experiences to seek reassurance, normalization, and niche support [14,15,40]. In addition, these videos may provide a platform for the posters to share and possibly validate their own infertility experiences.

Viewers also showed greater engagement with positive videos, which garnered more likes and comments than neutral videos. This may indicate viewer preference for videos that convey a positive outlook related to fertility. Video messages showing positive fertility outcomes may increase hope and optimism in the viewer, but they may also give a false impression of the success rates of fertility treatment. In actuality, less than half of embryo transfers for in vitro fertilization result in a live birth in the United States and Canada, with rates dropping with increasing maternal age [41,42]. Reliance on YouTube for infertility information may foster unrealistic expectations regarding the success rates of treatment, which may influence treatment decisions [38,43]. As fertility patients frequently access the internet for information [13,14], research is needed to establish whether YouTube content can affect the perceptions of infertility treatment among fertility patients.

In line with research suggesting that men are less likely to share YouTube videos [39], there was an absence of videos containing men only. When men were present, they were always accompanied by their female partner or other people (eg, friends or family). This finding is consistent with research showing that online activity and communication about infertility is more common among women [44-46]. Although we found a lack of male characters in videos, it remains possible that men seek out infertility content through other online avenues, such as websites and discussion forums. Research is needed to determine whether men use YouTube to search for information related to infertility or if they prefer other online platforms.

### Limitations and Future Directions

There are several limitations to the study and future directions to consider. As we only examined the content of high-rated videos, the results may not be generalizable to all infertility videos on YouTube. It is possible that less viewed and favored videos may also contain stories of negative experiences and treatment failures, which were mostly absent from highly viewed videos. YouTube videos with negative tones, such as sadness and anger, may be less viewed and preferred by viewers. Our study was a cross-sectional examination of videos and did not explore how video popularity changed over time. Due to the low number of videos where advice was offered to viewers, we were not able to assess quality of the information posted. Further research is needed to undertake such an assessment, as some studies have found health information on YouTube to be false or misleading [28,47,48]. Finally, as we analyzed publicly available content, we were only able to assess metrics available from YouTube and did not ask viewers their motivations for watching videos or the impact of these videos on their attitudes and beliefs. Future research should explore how infertile men and women search for videos, their reasons for doing so, and their reactions to the content of the videos.

### Conclusion

This analysis establishes highly viewed YouTube videos as a source of both technical and experience-based information related to infertility. Viewers appeared to prefer and engage more with videos containing personal experiences and positive tones. Although these videos may provide hope to viewers by frequently sharing positive treatment outcomes such as pregnancy announcements, they may also create and perpetuate unrealistic expectations about the success rates of fertility treatments.

## Abbreviations

IUI: intrauterine insemination
